# Biochar reshapes soil bacterial community composition and survival strategies: a meta-analysis revealing trade-offs between microbial stability and functional complexity

**DOI:** 10.1007/s00374-025-01971-9

**Published:** 2025-12-19

**Authors:** Jianwei Li, Jeewani H. Peduruhewa, Robert W. Brown, David R. Chadwick, Robert I. Griffiths, Haoran Fu, Hongfeng Bian, LianXi Sheng, Qinqxu Ma, Davey L. Jones

**Affiliations:** 1 State Environmental Protection Key Laboratory of Wetland Ecology and Vegetation Restoration, School of Environment, Northeast Normal University, Changchun, 130117 China; 2 School of Environmental & Natural Sciences, Bangor University, Bangor, LL57 2UW UK; 3 Zhejiang Provincial Key Laboratory of Agricultural Resources and Environment, College of Environmental and Resource Sciences, Zhejiang University, Hangzhou, 310058 China

**Keywords:** Biochar, Soil organic matter, Microbial communities, Survival strategies, Machine learning, Co-occurrence network

## Abstract

As a promising strategy for improving soil health and mitigating climate change, biochar has received increasing attention in recent years. However, findings regarding its microbial effects are often context-dependent, and the complex impacts of biochar on soil microbial communities remain inadequately understood. Here, we integrated 843 samples of 16 S rRNA sequencing data from 24 independent studies, utilizing machine learning and co-occurrence network analysis to evaluate general patterns and mechanisms by which biochar alters bacteria community composition and survival strategies. Overall, biochar significantly increased soil organic C, total N, pH, and reduced bulk density, which in turn affected microbial communities. While biochar addition did not notably alter bacterial α-diversity (Shannon index or species richness), it significantly changed β-diversity, indicating that biochar addition altered microbial community composition. Biochar changed community composition and survival strategies by reducing the oligotroph/copiotroph ratio, favouring copiotrophs groups (e.g., Bacteroidota). *K*-strategists (e.g., Planctomycetota) were reduced by biochar, potentially due to increased nutrient availability and increased competition. Model selection analysis identified that soil physicochemical properties were key drivers of α-diversity, while climate variables were the main factors influencing β-diversity. Biochar properties represented key factors influencing changes in microbial survival strategy. Machine learning identified 138 biomarker genera, with biochar-treated soils showing an increase in Pseudomonadota. Co-occurrence network analysis indicated that biochar increased microbial stability, but reduced network complexity, suggesting a trade-off between resilience and functional redundancy following biochar addition. These findings provide new insights into how biochar influences the composition and ecological functions of soil bacterial communities and offer theoretical support for its scientific application in sustainable soil management.

**Supplementary Information:**

The online version contains supplementary material available at 10.1007/s00374-025-01971-9.

## Introduction

Biochar, produced by pyrolyzing organic biomass under limited oxygen, is widely recognized as a promising soil amendment for improving fertility and supporting sustainable agriculture (Li et al. 2024; Waheed et al. 2025). Its stable aromatic structure and high surface area make it persistent in soil and effective in enhancing nutrient retention, water holding capacity, pH buffering, and heavy metal immobilization (Ding et al. 2016; Lehmann et al. 2011; Xu et al. 2023). These properties enable its application across diverse soil types and climates. Beyond improving soil physicochemical conditions, biochar also interacts with soil minerals and organic matter, influencing nutrient cycling and carbon stabilization (Ali et al. 2025). Importantly, it can alter soil microbial communities, especially bacteria that regulate key ecosystem functions (Palansooriya et al. 2019). Biochar owing to its porous structure, reactive surface groups and inert carbon framework has been widely adopted in agriculture and ecological restoration, where it reshapes microbial communities by altering soil pH, supplying labile carbon and increasing micro-habitat heterogeneity (Kracmarova-Farren et al. 2024). However, despite extensive research on biochar–microbe interactions, existing studies remain fragmented and primarily descriptive, and few have attempted to integrate microbial diversity, life-history strategies, and interaction networks into a unified mechanistic framework. This highlights the need for cross-system analyses that move beyond taxonomic responses alone.

Growing evidence shows that biochar enhances soil properties such as aeration, nutrient availability, and the quality of root soil interfaces (Palansooriya et al. 2025), all of which are known to regulate microbial community assembly. This implies that microbial responses to biochar are likely mediated by shifts in soil conditions, providing a useful framework for evaluating biochar effects across diverse studies. Soil microbial diversity is a key engine for terrestrial biogeochemical cycling, disease suppression and ecosystem resilience (Lehmann and Joseph 2015). The magnitude and direction of shifts in microbial diversity, however, depend strongly on the soil’s physicochemical background, local climate hydrology and application method. Alpha diversity refers to the richness and evenness within a single sample, while beta diversity reflects how community composition differs among samples. These components can respond differently to biochar-induced changes in soil resources and habitat composition, which helps explain the contrasting diversity patterns reported across different soil and climate conditions. In acidic or carbon-depleted croplands, moderate biochar additions raise pH and significantly enhance bacterial Shannon and Chao1 indices (Xu et al. 2023), whereas in long-term flooded or highly functionally redundant systems such as paddy fields and wetlands microbial communities are less responsive to exogenous carbon, and α-diversity can even decline (Dong et al. 2024; Xu et al. 2023). Biochar’s pore surface interfaces also steepen microscale gradients, increasing species turnover and thus β-diversity; such community shifts are often accompanied by enrichment of functional taxa like Bacillus and Pseudomonas, which bolster disease suppression and system resistance (Iacomino et al. 2022). Clarifying the synergistic or antagonistic effects of biochar on α- and β-diversity across diverse soil-climate scenarios is therefore essential for precision biochar deployment, sustainable agricultural productivity and resilient ecosystems. Although several meta-analyses have synthesized biochar effects on microbial α- and β-diversity, they have not examined how these taxonomic shifts relate to microbial ecological strategies or interaction-network stability, leaving important mechanistic questions unresolved.

The addition of biochar to soil can also alter the survival strategies of soil microorganisms (Chen et al. 2013; Zhang et al. 2017). This phenomenon is hypothesized to result from biochar’s ability to create a heterogeneous microenvironment, providing habitat niches, enhancing nutrient availability, and retention, and modifying soil pH and moisture conditions as well as supplying a small amount of labile soluble carbon (Campos et al. 2020; Jenkins et al. 2017; Jones et al. 2011). In particular, biochar’s porous composition (especially from lignocellulosic feedstocks) may serve as refugia for *r*-strategist bacteria, benefiting from the enhanced stability and nutrient retention provided by biochar (Yang et al. 2025), while some studies report that biochar benefits oligotrophic, *K*-strategist bacteria due to its porosity, surface area, and stable carbon inputs (Huang et al. 2024; Lehmann et al. 2011). The alteration of bacterial diversity resulting from biochar amendments is not only due to changes in community composition but also signifies a shift towards a more functionally stable and resilient microbial ecosystem that supports essential soil functions under environmental stresses (Campos et al. 2020). Microbial co-occurrence networks govern community robustness, functional redundancy, and disturbance resistance, yet the effects of biochar on their architecture and stability remain insufficiently resolved (Jones et al. 2011; Puente-Sánchez et al. 2024). Current studies indicate that biochar typically lowers connectome and increases modularity, thereby isolating niches and limiting disturbance propagation; it also induces new key bridge nodes, enhancing nutrient cycling and pathogen suppression (Huang et al. 2024) These topological shifts stem from biochar-driven increases in soil organic carbon (SOC) and decreases in bulk density (BD), which improve aeration, water retention, and positive interactions (Ding et al. 2016; Lehmann et al. 2011; Xu et al. 2023). SOC and BD are regulated by feedstock type, pyrolysis temperature, and application rate, factors that determine the balance between *r*- and *K*-strategists and thus mediate the net effect of biochar on network stability (Yang et al. 2025). Clarifying the coupled mechanisms among biochar-altered soil properties, microbial life-history strategies, and network topology is critical for the precise application of biochar to strengthen nutrient retention and ecosystem resilience. Although individual studies suggest that biochar may influence microbial survival strategies through changes in resource availability and habitat heterogeneity, these patterns have not been evaluated at a global scale, and it remains unclear whether such shifts represent consistent biochar-mediated ecological responses.

Integrative meta-analyses of sequencing data are essential for identifying general trends, context-specific responses, and underlying ecological processes in biochar-amended soils (Jenkins et al. 2017). This requires advanced bioinformatic frameworks capable of synthesizing large, heterogeneous datasets. Recent advances in high-throughput technologies, particularly 16 S rRNA gene amplicon sequencing, have deepened our understanding of biochar’s effects on soil bacterial communities (Hua et al. 2021; Xu et al. 2016), revealing mechanisms that support microbial diversity and ecosystem function (Jenkins et al. 2017; Lehmann et al. 2011). However, the rapid expansion of sequencing data poses challenges in cross-study comparability, standardization, and integration across varying experimental conditions (Ten Hoopen et al. 2017). Unlike previous meta-analyses that relied mainly on reported summary metrics, our study reprocessed the original 16 S rRNA sequencing data from each experiment using a unified bioinformatic pipeline. This approach minimizes methodological inconsistencies across studies and allows a more mechanistic evaluation of microbial survival strategies, biomarker taxa, and network stability under biochar amendments. In this study, we conducted a meta-analysis of 16 S rRNA gene sequencing data to investigate how biochar amendments affect soil bacterial diversity, ecological strategies, and community stability. We hypothesized that (1) biochar does not consistently increase bacterial α-diversity due to community saturation and functional redundancy; (2) biochar preferentially enriches *r*-strategist (copiotrophic) taxa due to enhanced nutrient availability and labile carbon inputs; and (3) changes in microbial community stability are primarily influenced by BD and SOC. By linking bacterial composition, diversity, and inferred ecological strategies to biochar-altered soil properties, this study provides a novel integrative framework for understanding the microbial mechanisms driving biochar-induced shifts in community stability and ecological function.

## Materials and methods

### Data collection and filtering

A systematic literature search was conducted using Web of Science, Google Scholar, and China National Knowledge Infrastructure to identify peer-reviewed studies published between January 1, 2016, and December 1, 2024, a period corresponding to the widespread adoption of high-throughput sequencing technologies. The search followed PRISMA guidelines (Moher et al. 2009; Fig. S1), and the checklist is provided as a separate supplementary file. The search strategy used keyword combinations such as (biochar OR biochar amendment OR biochar soil addition) AND (bacterial community OR microbial diversity OR microbiome) AND (16 S rRNA OR high-throughput sequencing OR amplicon sequencing), resulting in 1,142 initial records. Only bacterial 16 S rRNA datasets were included in the final analysis. Studies were included if they: (i) focused on biochar-amended soils with control and treatment comparisons; (ii) targeted the 16 S rRNA gene (V3-V4 or V4 region) using standard primers (e.g., 338 F/806R, 515 F/806R); (iii) used high-throughput sequencing platforms (e.g., Illumina MiSeq/NovaSeq); and (iv) provided metadata on biochar characteristics and experimental design. Studies involving farmyard manure or lacking field-based in situ sampling were excluded to avoid confounding effects. To ensure comparability, only trials where biochar was the primary treatment and other nutrient inputs were absent or consistently applied were selected. Metadata were retrieved from public databases (e.g., National Center for Biotechnology Information Sequence Read Archive, European Nucleotide Archive) and included soil type, climate, cropping system, sequencing details, and experimental parameters. After screening, 24 studies comprising 843 samples were retained for analysis (Data S1; Fig. S1). Detailed information on metadata, such as biochar type and crop species, is provided in Data S1. We obtained 843 data points from 24 independent studies. The study area was primarily confined to the Northern Hemisphere, primarily in regions of Asia, with mean annual temperatures ranging from 4.5 to 23.1 °C (Data S1, Fig. S2). The predominant soil types are Mollisol and Luvisol. The primary source of biochar was cereal straw, accounting for 64% of biochar feedstock in the studies included. The predominant planting practice was cropping rotation. The average biochar application rate was 36.92 Mg/ha, with a range from 0.8 Mg/ha to 90 Mg/ha, and the average application duration was 2.66 years, with a range from 0.25 years to 10 years.

### Bioinformatics analysis

All raw 16 S rRNA FASTQ files were retrieved from public repositories and reprocessed using a unified QIIME2 (Bolyen et al. 2019) workflow to minimize methodological inconsistencies among studies. Sequence quality was first assessed with FastQC, and primers were removed using Cutadapt (Martin 2011). Paired-end reads were merged with VSEARCH (Rognes et al. 2016), and low-quality bases (Phred < 20) were filtered using QIIME2 default parameters. Amplicon sequence variants (ASVs) were denoised with Deblur following Comeau et al. (2017) and Wright et al. (2021), using study-specific trimming lengths determined from quality profiles. For several datasets in which reverse reads were of insufficient quality, only forward reads were retained, consistent with procedures used in recent meta-analyses. Taxonomic assignment was performed using the SILVA v132 reference database. Samples with fewer than 2000 reads and ASVs with cumulative abundance ≤ 10 were removed, after which all datasets were rarefied to 2000 reads per sample to correct for large differences in sequencing depth across studies. Feature tables and representative sequences were merged using QIIME2’s feature-table merge commands, yielding a harmonized dataset of 843 samples from 24 studies. Due to substantial variability in amplicon sequence variants (ASV) composition across studies, we focused primarily on genus-level analyses, excluding unannotated sequences to preserve taxonomic diversity and enhance cross-study comparability (Chong et al. 2020). Genus-level profiles were generated by aggregating ASVs assigned to the same genus based on taxonomic annotation. ASV-level analyses were also conducted, with low-prevalence ASVs (present in < 10% of samples) removed to minimize technical noise and avoid richness overestimation. Notably, ASV-level classification did not improve the discrimination between control and biochar-treated groups (Table S3), aligning with previous findings (Lei et al. 2023; Yuan et al. 2020). Bacterial life strategies were categorized according to the phylum level with, Acidobacteriota, Actinomycetota, Planctomycetota and Chloroflexota classified as likely oligotrophic bacterial taxa (*K*-strategists), and Bacillota, Gemmatimonadota, and Bacteroidota as classified as copiotrophic taxa (*r*-strategists) (Hu et al. 2023; Li et al. 2021). Pseudomonadota (Proteobacteria), being a highly diverse phylum with different subgroups exhibiting distinct ecological strategies, were therefore not included in this classification. The rrnDB database was used to estimate the number of rrn copies of each ASV abundance-weighted, and the rrn copy number was used to determine whether the microbes were *K*-strategists or *r*-strategists (Hu et al. 2023; Li et al. 2023). Correlation analysis revealed a significant negative relationship (*p* < 0.01) between rrn operon copy number and the oligotrophs/copiotrophs ratio (Fig. S3). This pattern suggests that growth-strategy tendencies inferred from rrn traits are aligned with those observed at both the phylum level and the whole-community scale.

### Environmental data extraction

Climate data (30 arc-second resolution), including mean annual temperature (MAT) and precipitation (MAP), were obtained from the WorldClim database (Fick and Hijmans 2017), and soil type data from ISRIC (https://www.isric.org/). Soil properties (pH, SOC, TN, BD) were extracted directly from study tables or digitized from figures using GetData Graph Digitiser (v2.24). Crop types and cropping systems were also recorded to support modeling of land-use effects. In addition, the sample collection time reported in each study was recorded to account for potential seasonal or temporal influences on soil microbial communities. Variables were selected based on availability and consistency across studies. Less frequently reported variables, such as total phosphorus, were excluded to minimize data loss. Metadata on biochar characteristics (feedstock type, pyrolysis temperature, application rate, and duration) were included as covariates in model selection analyses to account for variability among biochar.

### Statistical analyses

To reduce dimensionality and improve model performance, bacterial data were aggregated at the genus level and analyzed using relative abundances. All analyses were conducted in R (v4.3.2). A random-effects model was fitted using the lme function from the nlme package (Pinheiro 2011), with α-diversity and survival strategies as response variables, and biochar application rate and duration as fixed effects; study identity was included as a random effect. Group differences were tested using the Mann-Whitney U test. Genus-level data were used to assess β-diversity via Bray-Curtis dissimilarity and visualized with PCoA. PERMANOVA (999 permutations) was performed using the vegan package (Oksanen 2024) to test treatment effects. The top 10 bacterial phyla were used to evaluate compositional shifts.

As this study integrates 24 datasets spanning diverse soil types, climatic conditions, and experimental environments, machine learning served as an effective approach for identifying taxa that showed consistent and ecologically meaningful responses to biochar across studies. Therefore, we applied three machine learning classifiers Random Forest, Support Vector Machine, and Logistic Regression to ASV relative abundances to further distinguish bacterial community composition between biochar-treated and control samples. All models were trained and evaluated using an 80/20% training test split with five-fold cross-validation, and feature selection was performed using LASSO followed by recursive feature elimination (rfcv) with 500 trees. Model performance was assessed using ROC curves and AUC values. Detailed procedures, parameter settings, and robustness tests are provided in Supplementary Methods (Text S2). To assess bacterial metabolic functions related to nutrient cycling, functional annotation was performed using the FAPROTAX database (Louca et al. 2016). Community assembly processes were evaluated using the Neutral Community Model (NCM; Burns et al. 2016) and the normalized stochasticity ratio (NST; Ning et al. 2019), with NST > 0.5 indicating stochastic dominance. Bacterial association networks were constructed using Spearman correlations (|r| > 0.4, *p* < 0.05), calculated with the Hmisc package and corrected using the Benjamini-Hochberg method. Network topology (Yuan et al. 2020) was assessed using a composite complexity index that integrated normalized average degree, modularity, clustering coefficient, and inverse diameter/path length. To evaluate ecological stability, we used two complementary metrics. Structural stability was quantified as network robustness, defined as the proportion of taxa retained after random removal of 50% of the nodes, and this process was monitored through changes in natural connectivity (Yuan et al. 2021). Compositional stability was assessed using the average variation degree AVD, which measures fluctuations in the relative abundance of taxa among samples. Lower AVD values indicate a more stable community composition. Together, robustness and AVD describe complementary dimensions of stability and help explain why an increase in network complexity does not always result in greater resilience. Networks with dense linkages or strong modular organization can transmit disturbances more effectively, which reduces structural robustness even when interaction complexity appears high. Although these two indicators cannot capture every aspect of ecological resilience, they are widely used and provide a useful and complementary framework for evaluating the reorganization of microbial interaction networks following biochar amendment. To account for sample size differences (Biochar: 517; Control: 326), two network sets were constructed: one using all samples and another using 300 randomly selected samples (resampled 100 times). We conducted model selection analysis to identify key factors influencing bacterial diversity, community composition, and survival strategies under biochar amendment. Variable importance was assessed using the sum of Akaike weights across all models, with a threshold of 0.8 used to define significant predictors (Terrer et al. 2016; Wang et al. 2025). Analyses were performed using the glmulti package (Calcagno and Mazancourt 2010). To explore direct and indirect effects of biochar and soil properties on bacterial diversity, we used piecewise structural equation modeling (SEM) fitted via linear mixed-effects models, treating study identity as random factors (Fig. S5). An initial full model was simplified by removing non-significant paths, and final model fit was assessed using a Chi-squared test in the piecewiseSEM package (Lefcheck 2016). Code is available at: https://github.com/uby76/Biochar.

## Results

### Effect of biochar addition on microbial diversity

**Fig. 1:**
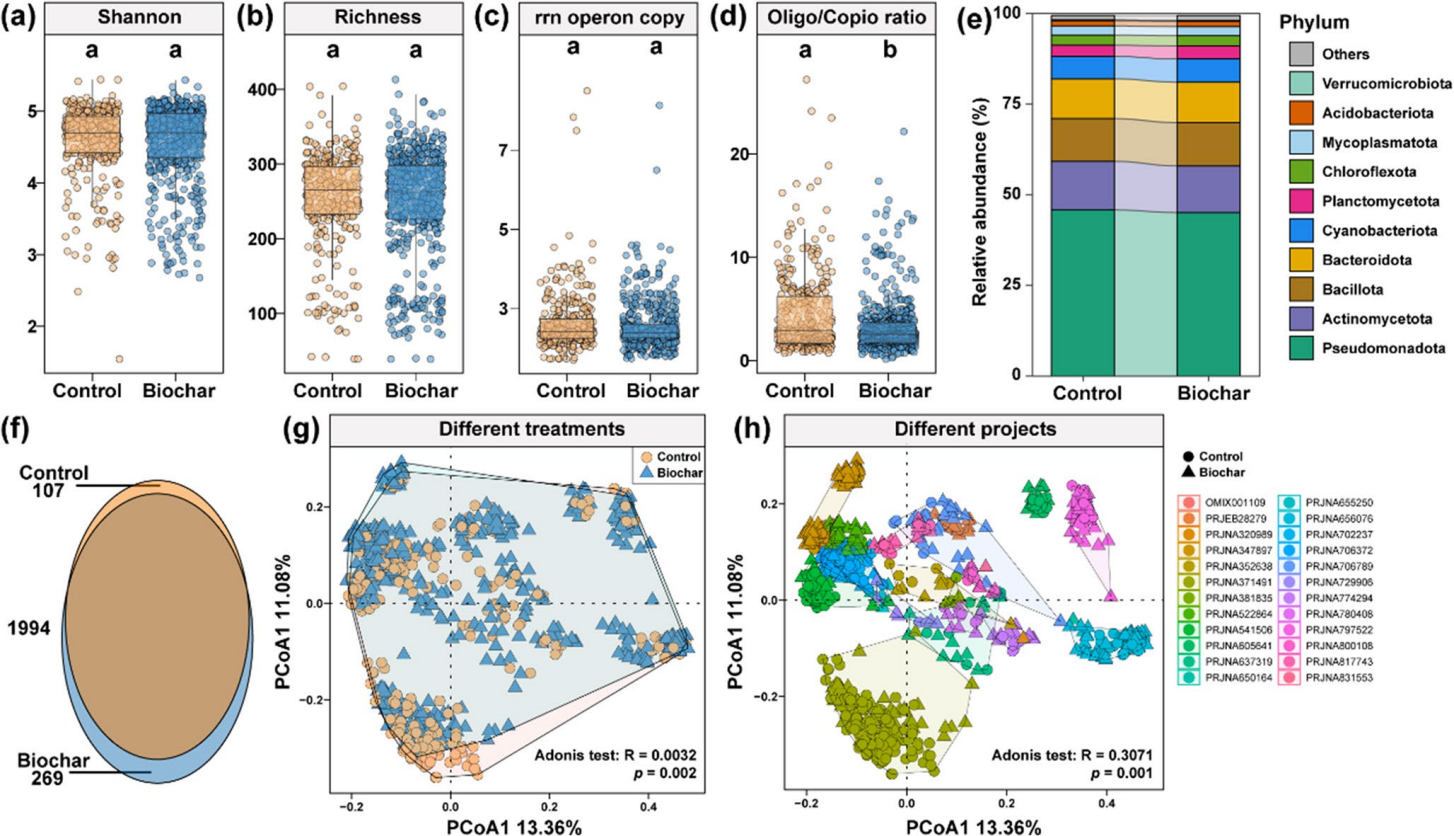
Effects of biochar application on soil bacterial community diversity, composition, and functional strategies. (**a**, **b**) Bacterial α-diversity, assessed using the Shannon index and species richness, for all samples, including 326 from the control group and 517 from the biochar group. The horizontal line in each boxplot (**a**, **b**, **c**, **d**) represents the median, while the top and bottom edges indicate the 75th and 25th percentiles, respectively. (**c**, **d**) Bacterial survival strategies, measured using two approaches: (**c**) the mean copy number of 16 S rRNA genes (rrnDB) within the bacterial community and (**d**) the ratio of oligotrophs to copiotrophs bacteria (Oligo/Copio ratio). (**e**) Relative abundance (%) of major bacterial phyla in the control and biochar-treated groups. (**f**) Venn diagram illustrating the number of shared and unique genus between the control and biochar groups. (**g**, **h**) Principal Coordinate Analysis (PCoA) based on Bray-Curtis similarity, visualizing bacterial community differences: (**g**) based on treatment effects and (**h**) based on data sources. All reported significance values were obtained using the Mann-Whitney U test, with different studies treated as random effects to assess whether there were significant differences between the control and biochar treatments

**Fig. 2:**
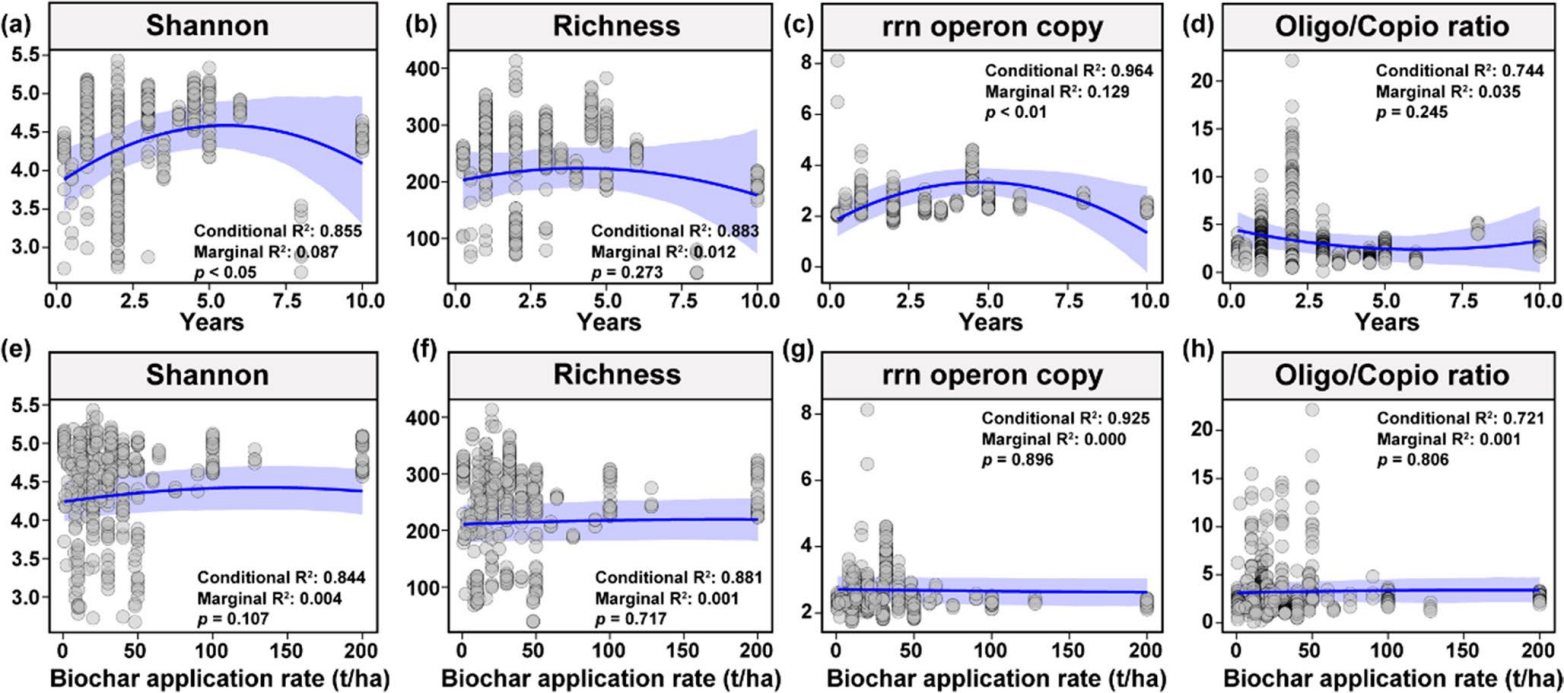
Effects of biochar application duration and application rate on soil microbial diversity and life-history strategies based on linear mixed-effects models (different projects as random effects). rrn operon copy represents the average 16 S rRNA gene copy number (rrnDB) within the bacterial community, Oligo/Copio ratio denotes the ratio of oligotrophs to copiotrophs bacteria

Following biochar application, soil nutrient levels significantly increased: SOC rose from 17.93 to 48.21 g/kg (*p* < 0.05), and TN from 1.81 to 1.93 g/kg (*p* = 0.061; Fig. S4a-b). Soil pH increased from 6.35 to 6.90 (*p* < 0.05), while bulk density (BD) decreased from 1.31 to 1.25 g/cm³ (*p* < 0.01; Fig. S4c-d). However, biochar did not significantly affect bacterial α-diversity, as measured by the Shannon index, species richness, or rrnDB copy number (Fig. 1a-c). Notably, biochar significantly reduced the oligotroph/copiotroph ratio (Fig. 1d) and decreased the relative abundance of Planctomycetota (*p* < 0.05; Fig. 1e, Table S1). Beta diversity analysis (Bray-Curtis PCoA) revealed significant shifts in bacterial composition between treatments (*p* < 0.01, PERMANOVA; Fig. 1g-h). Biochar-treated soils also harbored more unique bacterial genera (269 vs. 107; Fig. 1f). To further assess diversity responses, linear mixed-effects models were used to evaluate the influence of application duration and rate (Fig. 2). Shannon diversity and rrnDB copy number followed a non-linear trend with duration increasing during the first five years (*p* < 0.05 and *p* < 0.01, respectively), then declining (Fig. 2a and c), indicating a temporal shift towards *r*-strategists. In contrast, biochar application rate had no significant effect on bacterial diversity (Fig. 2e-h).

### Key environmental predictors and microbial biomarkers shaped by biochar

**Fig. 3:**
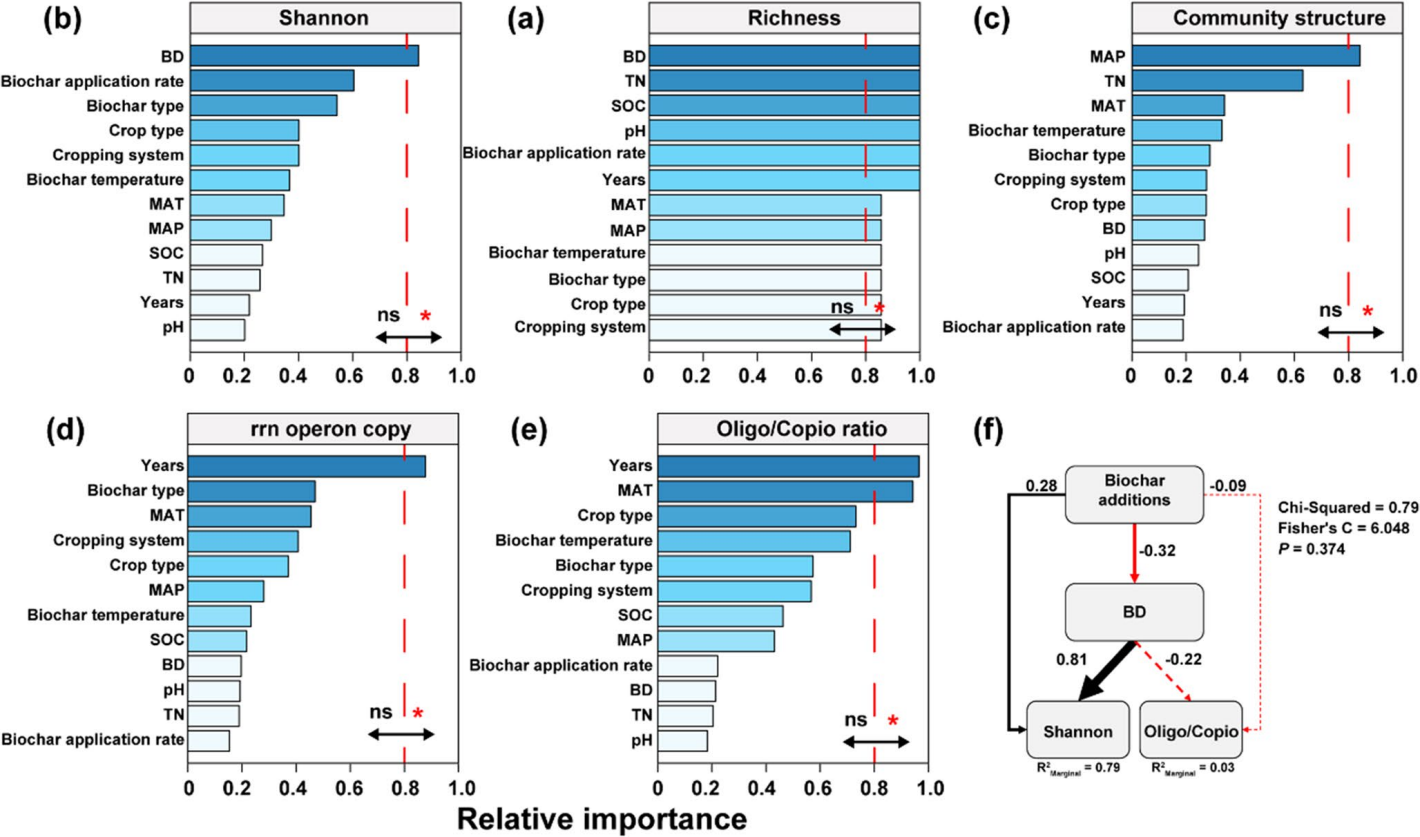
The relative importance of biochar application on bacterial α-diversity, β-diversity, and microbial survival strategies was assessed based on the sum of Akaike weights derived from model selection using the corrected Akaike Information Criterion (AICc), with a threshold of 0.8 set to distinguish key predictors from non-essential factors (**a**-**e**). Structural equation modeling showing the effects of biochar addition and bulk density on Shannon diversity and survival strategies. Black and red arrows indicate significant positive and negative effects, respectively (*p* < 0.05), while dashed arrows indicate nonsignificant relationships. Values next to the arrows represent standardized path coefficients. The width of the arrows is proportional to the strength of the path coefficients. The coefficient of determination (R^2^) represents the proportion of variance in the dependent variable that is explained by the independent variable(s) in the model. rrn operon copy represents the average 16 S rRNA gene copy number (rrnDB) within the bacterial community, Oligo/Copio ratio denotes the ratio of oligotrophs to copiotrophs bacteria. Years: duration of biochar application; MAT: mean annual temperature; MAP: mean annual precipitation; SOC: soil organic carbon content; BD: bulk density; TN: total nitrogen

We employed model selection analyses to identify key environmental and climatic factors influencing soil microbial community diversity. Specifically, BD emerged as the primary drivers of α-diversity (Fig. 3a and b), while MAP influenced community composition (PCoA1, Fig. 3c). Notably, the duration of biochar application had a significant impact on changes in *r*/*K* selection strategies, as indicated by the average copy number of 16 S rRNA genes and the Oligo/Copio ratio (Fig. 3d and e). To further explore the underlying mechanisms, SEM was utilized to assess how biochar application affected soil microbial diversity and survival strategies (Fig. 3f). Biochar application significantly reduced soil bulk density (covariance coefficient = -0.32, *p* < 0.05), and the reduction in bulk density drove changes in Shannon (covariance coefficient = 0.81, *p* < 0.05); however, biochar application rate and changes in bulk density did not drive changes in bacterial survival strategies.

**Fig. 4:**
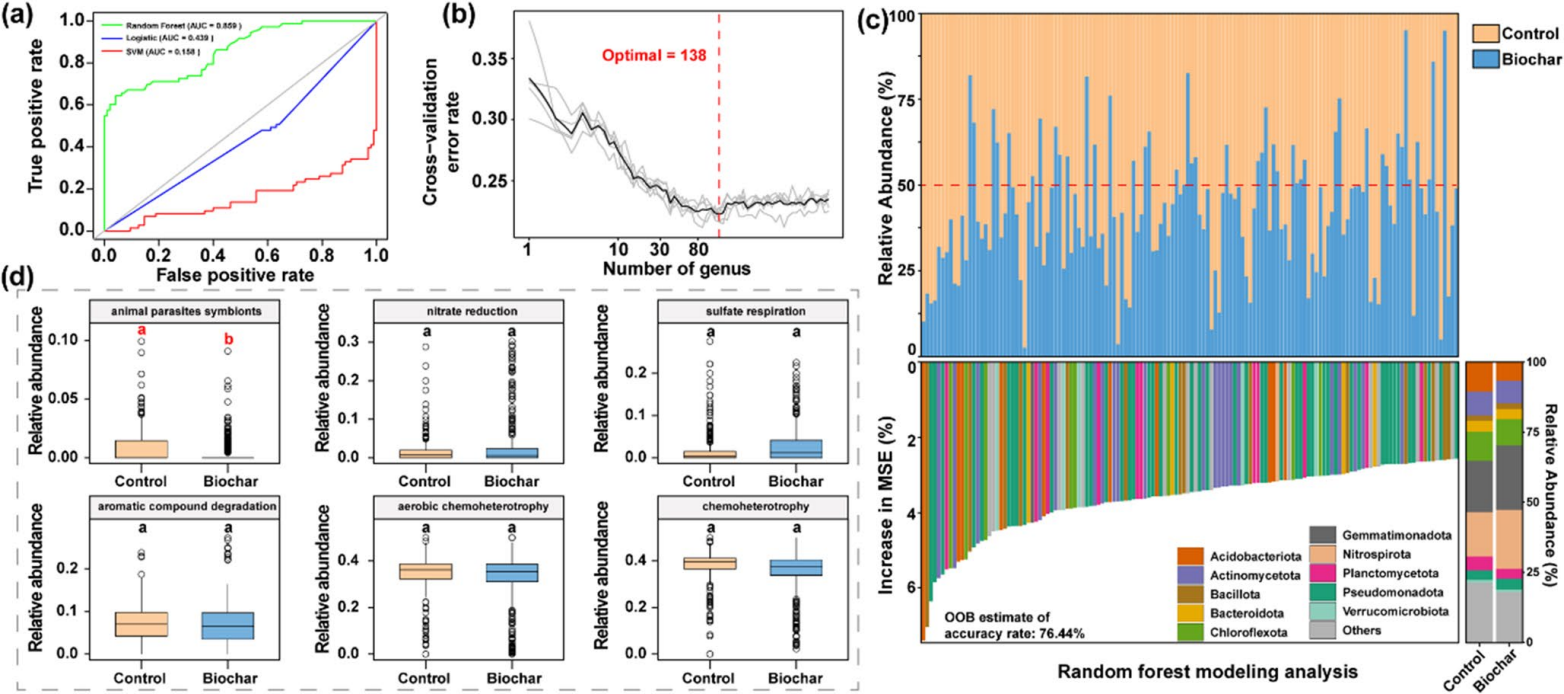
Identification of biomarker microbial communities and functional predictions using machine learning. (**a**) The dataset consists of 326 control samples and 517 biochar-treated soil samples, which were used to construct classification models with three machine learning algorithms: random forest (RF), support vector machine (SVM), and logistic regression (LR). The area under the curve (AUC) values for these models at the genus level are shown, with the RF model achieving the highest classification accuracy (AUC = 0.859). (**b**) A random forest model was constructed at the genus level, and the relationship between five-fold cross-validation (CV) error and the number of genera was evaluated. During this process, the out-of-bag (OOB) error was calculated to assess model performance, identifying 138 key genera that differentiate control and biochar-treated soils. (**c**) The relative abundance changes of the 138 genera were analyzed between the control and biochar-treated groups, alongside the taxonomic composition at the phylum level. (**d**) The relative abundance variations of these 138 genera were further examined within six functional groups predicted using the FAPROTAX database. All reported significance values were obtained using the Mann-Whitney U test, with different studies treated as random effects to assess whether there were significant differences (*p* < 0.05) between the control and biochar treatments

Among these models, the random forest model demonstrated the best performance (AUC = 0.859, Fig. 4a). Therefore, the random forest model was selected to identify biomarkers at the bacterial genus level. To prevent overfitting, a 10-fold cross-validation with five replications was conducted. The cross-validation error curves stabilized when selecting 138 genus-level taxa as biomarkers (Fig. 4b). The most important biomarker genera were predominantly affiliated with the phyla Gemmatimonadota and Nitrospirota, while control samples were enriched in Chloroflexota, Planctomycetota, and Verrucomicrobiota (Fig. 4c, Table S2). Additionally, biochar-treated soils exhibited a significant enrichment of Pseudomonadota (*p* < 0.05). Functional predictions using the FAPROTAX database revealed that biochar application significantly reduced the relative abundance of animal parasite symbionts (*p* < 0.05) in the soil (Fig. 4d).

### Effects of biochar on microbial community assembly and network complexity

**Fig. 5:**
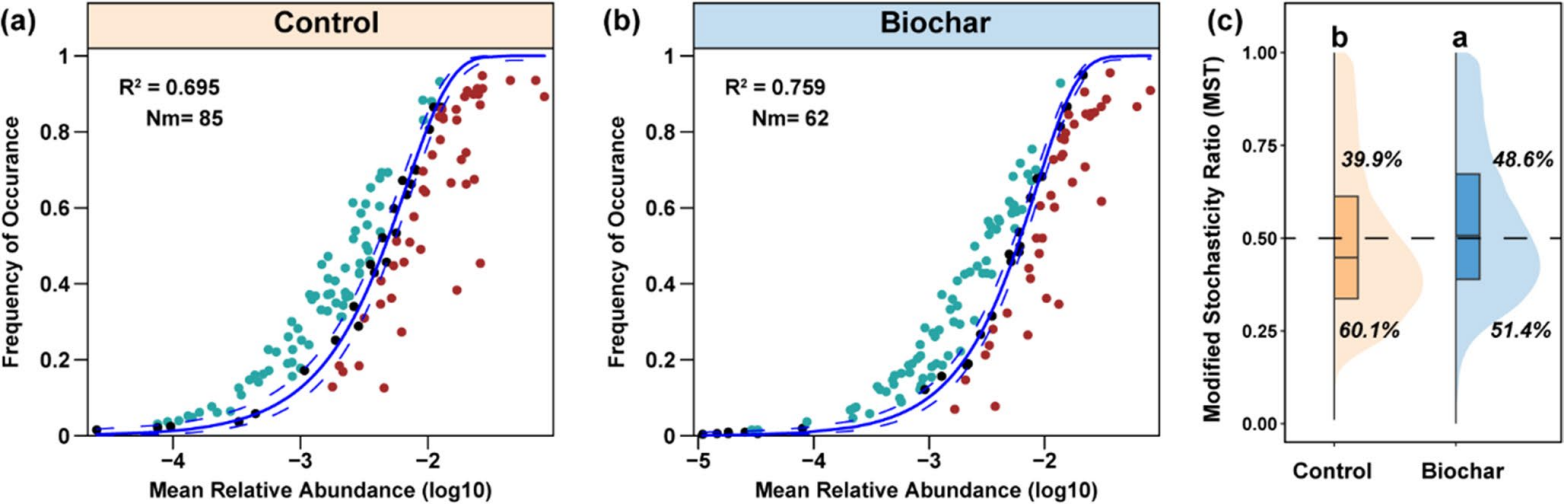
Mechanisms of community construction for microbial diversity patterns under control and biochar addition. (**a**, **b**) Neutral Community Model (NCM) fitted separately for the control and biochar groups, predicting the observed occurrence frequency of microbial genera. The solid blue line represents the best-fit NCM, while the dashed blue lines indicate the 95% confidence interval. Genera whose observed occurrence frequencies deviate significantly from the predicted values are highlighted in distinct colors. The parameter Nm represents the product of metacommunity size and migration rate, while coefficient of determination (R^2^) quantifies the model’s goodness-of-fit. (**c**) Normalized Stochasticity Ratio (NST), which evaluates the relative contribution of stochastic processes in microbial community assembly. The numbers within the plot denote the proportion of NST values greater than 0.5 and less than 0.5, indicating the dominance of stochastic or deterministic assembly processes, respectively. Statistical significance was assessed using the Mann-Whitney U test

**Fig. 6:**
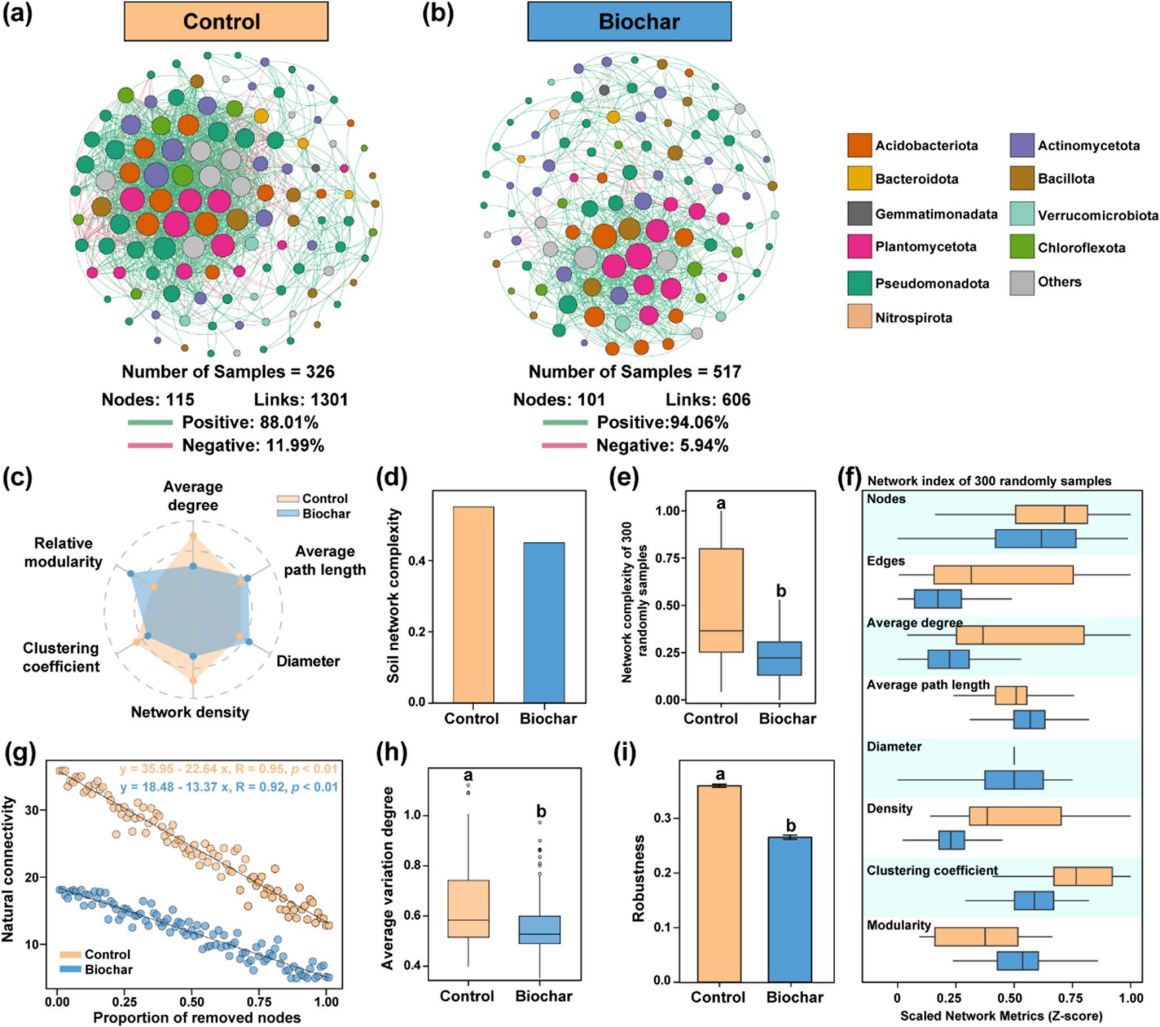
Network and community stability analysis of biomarker taxa in the control and biochar treatment groups. (**a**, **b**) Co-occurrence networks of soil microbial communities in the control and biochar treatment groups, where |r| > 0.4, *p* < 0.05. Different colors represent bacterial phyla at different taxonomic levels. (**c**) Radar plot illustrating the normalized differences in network topological features between treatments. (**d**) Network complexity analysis, in which topological parameters were summed to assess the overall complexity of soil microbial networks. The reciprocal of the average path length and network diameter was computed to evaluate network connectivity. (**e**) A subset of 300 microbial taxa was randomly selected from the control and biochar groups to construct co-occurrence networks, followed by the calculation of network complexity parameters. (**f**) Standardized values of various network indices in randomly generated networks for the control and biochar treatments. (**g**) Robustness analysis depicting the relationship between natural network connectivity and node removal proportion. Higher node removal proportions indicate lower soil network stability. (**h**) Community mean variability analysis comparing microbial community stability between the control and biochar treatments. Higher values indicate lower microbial community stability. (**i**) Robustness assessment measuring the proportion of retained taxa after randomly removing 50% of the taxa in the control and biochar treatments. Error bars represent the standard error from 100 simulation replicates. All reported significance values were determined using the Mann-Whitney U test

A neutral community model based on 138 key bacterial taxa (identified via random forest) showed that biochar application suppressed bacterial succession, reducing metacommunity size and migration rate (Nm: 85 to 62), while increasing stochasticity (Fig. 5a-b). NST analysis further indicated a higher proportion of stochastic assembly in the biochar group (48.6%) than in the control (39.9%; *p* < 0.01; Fig. 5c). Alpha diversity (Shannon, Richness) was significantly higher in the control group, while β-diversity was lower in the biochar group (Fig. S6). Co-occurrence networks constructed from the 138 taxa revealed reduced nodes and edges under biochar treatment, but a higher proportion of positive correlations (94.1% vs. 88.0%; Fig. 6a–b). Network complexity was consistently greater in the control group, even after sample size normalization (Fig. 6d-f). AVD results showed higher microbial community stability in the biochar group (Fig. 6h, *p* < 0.01), whereas natural connectivity and robustness analyses indicated higher network stability in the control group (Fig. 6i). Key taxa in the biochar network primarily belonged to Planctomycetota, Bacillota, Cyanobacteriota, and Pseudomonadota, with relative abundances showing a decline–rebound trend over time (Fig. S7a-b, S8).

**Fig. 7:**
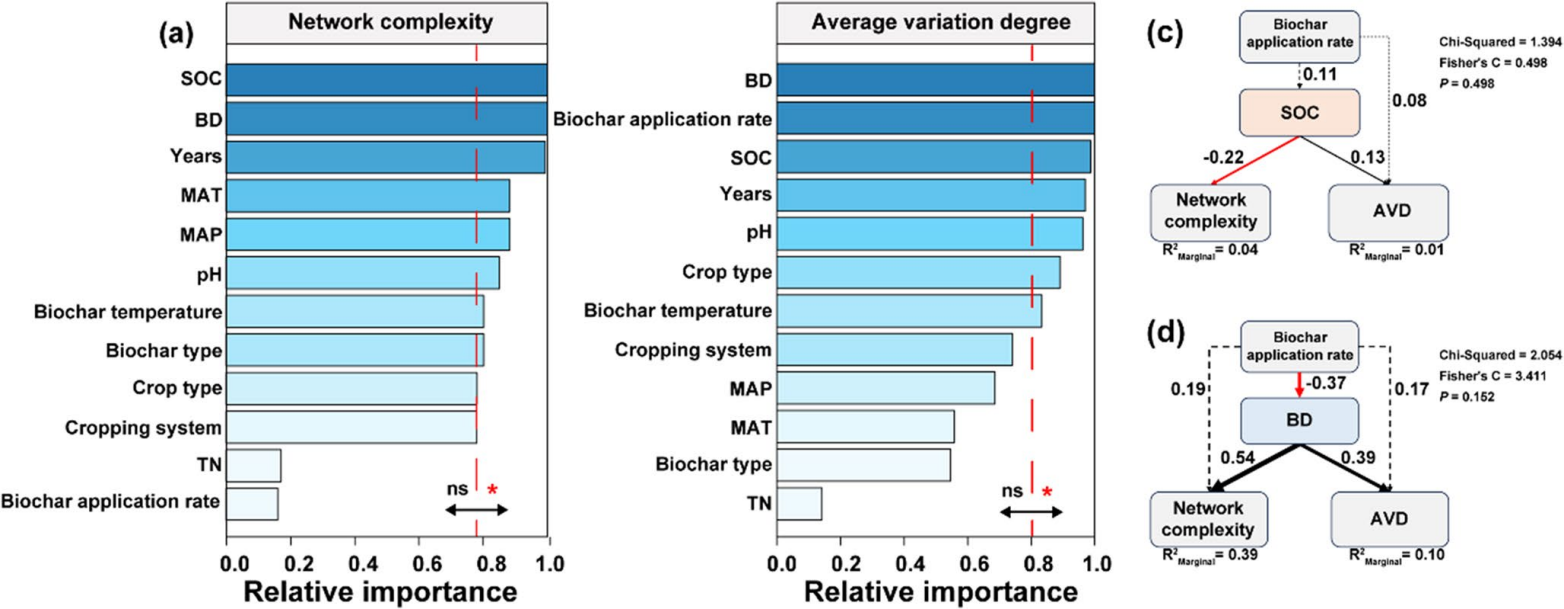
Relative importance of biochar application on bacterial network complexity and average variation degree was assessed based on the sum of Akaike weights derived from model selection using the corrected Akaike Information Criterion (AICc), with a threshold of 0.8 set to distinguish key predictors from non-essential factors (**a** and **b**). Structural equation modeling (SEM) showing the effects of biochar addition, soil organic carbon content and soil bulk density on network complexity and average variation degree (**c** and **d**). Black and red arrows indicate significant positive and negative effects, respectively (*p* < 0.05), while dashed arrows indicate non-significant relationships. Values next to the arrows represent standardized path coefficients. The width of the arrows is proportional to the strength of the path coefficients. The coefficient of determination (R^2^) represents the proportion of variance in the dependent variable that is explained by the independent variable(s) in the model. Average variation degree (AVD) quantifies the temporal or spatial variability of community composition, with higher values indicating lower stability. Years: duration of biochar application; MAT: mean annual temperature; MAP: mean annual precipitation; SOC: soil organic carbon content; BD: bulk density; TN: total nitrogen

Model selection analyses were used to identify the key factors influencing bacterial network complexity and stability. The analysis revealed that soil nutrients play a critical role in shaping bacterial interactions, with SOC and BD identified as a primary driver of microbial network complexity and stability (Fig. 7a and b). SEM was employed to explore the relationship between biochar application and bacterial network properties. The increase in SOC led to a reduction in bacterial network complexity and an enhancement of microbial stability (Fig. 7c). Increasing biochar application significantly reduced soil BD (covariance coefficient = -0.37, *p* < 0.05) (Fig. 7d). Additionally, higher BD was associated with increased stability of both the bacterial network and community (Fig. 7d).

## Discussion

### Effect of Biochar on alpha diversity and species composition of soil bacteria

Growing evidence indicates that the response of microbial α-diversity to biochar is strongly governed by underlying soil physicochemical properties. Biochar mainly alters soil composition, pH, and nutrient availability (Palansooriya et al. 2025), and such soil-mediated changes often shape microbial processes more strongly than diversity metrics themselves. Consistent with this broader understanding, biochar addition did not significantly affect soil bacterial α-diversity (Shannon index and richness; Fig. 1a and b), supporting hypothesis 1. This lack of response may reflect the ecological stability and functional redundancy of microbial communities and their ability to buffer environmental change (Jenkins et al. 2017; Nunan et al. 2020). Although biochar improves soil physicochemical properties such as porosity and nutrient availability it appears that soil microbial communities are resilient and can adapt to such environmental changes. This result is consistent with the broader understanding that soil physicochemical properties are the major regulators of microbial diversity, often exerting stronger control than management interventions such as biochar addition. Our model selection analysis also supports this view, indicating that bulk density was the key predictor of α-diversity across studies. Therefore, the weak response of α-diversity to biochar likely reflects a balance between improved soil conditions and the inherent ecological buffering capacity of microbial communities. Instead of altering total diversity, biochar primarily influenced community functional strategies through nutrient enrichment, leading to the proliferation of copiotrophic taxa and a reduction in oligotrophs. Previous studies have similarly shown that biochar does not consistently increase microbial α-diversity, largely because microbial responses are mediated by existing soil conditions, resource gradients, and the traits of resident taxa rather than by biochar alone (Delgado-Baquerizo et al. 2016; Lei et al. 2023; Li et al. 2020). Nevertheless, biochar addition profoundly influences bacterial survival strategies (Bolan et al. 2023). Rather than directly increasing bacterial species Richness, biochar modifies the soil environment, driving microbial community adaptation (Xu et al. 2023). Biochar improves soil physicochemical properties, such as increasing SOC and TN content while altering soil water retention capacity and pH levels (Fig. S4). While the rrnDB gene copy number remained unchanged, the oligotrophs/copiotrophs ratio decreased, suggesting that biochar amendments enhance soil resource availability, favoring the proliferation of copiotrophs bacteria (Fig. 1c and d). Biochar-enhanced nutrient availability can create resource-rich microsites that disproportionately benefit fast-growing, resource-acquisitive taxa. The shift toward copiotrophic bacteria observed here is consistent with reports that biochar increases nutrient availability and improves rhizosphere resource conditions (Palansooriya et al. 2025), thereby promoting *r*-strategist microbial groups. This shift aligns with *r*/*K* selection theory, indicating a transition from *K*-strategist microbes (slow-growing, resource-efficient organisms) to *r*-strategists (rapidly growing microbes that respond swiftly to environmental changes). This is consistent with our hypothesis 2, which posits that biochar application alters microbial community functional strategies by favoring copiotroph (*r*-strategist) bacteria over oligotroph (*K*-strategist) taxa due to enhanced soil nutrient availability. As a result, high-metabolism microbes gained a competitive advantage, while the relative abundance of *K*-strategist bacteria declined (Stone et al. 2023). This phenomenon likely stems from biochar’s ability to optimize soil nutrient retention and release, thereby increasing available carbon sources and nutrients, which in turn benefit fast-growing bacterial taxa (Hu et al. 2023). It is important to note, however, that *r/K* (oligotroph/copiotroph) pattern reflect broad ecological tendencies rather than strict functional classifications, and should therefore be interpreted with appropriate caution.

Significant changes in β-diversity and the decreased relative abundance of taxa like Planctomycetota indicate microbial community restructuring following biochar application (Fig. 1e). This likely results from altered microbial competition, where fast-growing, nutrient-responsive *r*-strategists outcompete slower-growing *K*-strategists. Planctomycetota, typically *K*-strategists with high NH₄⁺ uptake capacity (Stephenson et al. 2024), may have declined due to reduced NH₄⁺ availability from biochar adsorption. Concurrently, increased SOC and dissolved organic carbon (Chagas et al. 2022) favored *r*-strategists such as Bacteroidota. Structural equation modeling confirmed that soil bulk density was a key driver of bacterial shifts, supporting hypothesis 3. This suggests biochar indirectly influences bacterial communities by altering soil conditions and promoting niche adaptation. Linear mixed-effects models showed that Shannon diversity and rrnDB copy number increased during early application years and declined later (Fig. 2), though interpretation is limited by the scarcity of long-term data (> 5 years). Crop type and cropping system were also tested in the SEMs but did not significantly improve explanatory power (Fig. 3), possibly due to limited data coverage or insufficient detail in reported practices.

### Effects of Biochar addition on core microbial communities

This study systematically analyzed the effects of biochar on soil bacterial communities using machine learning techniques and identified key biomarkers core bacterial taxa to explore regulatory mechanisms. Biochar application significantly altered bacterial community composition and functional traits. Pseudomonadota, a metabolically active phylum containing many taxa involved in carbon degradation, nitrogen cycling, and stress tolerance, was enriched in biochar-treated soils, whereas Chloroflexota, Planctomycetota, and Verrucomicrobiota were more abundant in the controls (Fig. 4c). This trend suggests biochar may promote *r*-strategist microbes and reduce the competitiveness of *K*-strategists by modifying soil pH, carbon availability, and oxygen diffusion (Shang et al. 2023). Although biochar primarily exerts abiotic effects (e.g., pH, nutrients, oxygen), plant-mediated influences such as altered root exudation should not be overlooked. However, statistical analysis showed no significant effects of crop type or cropping system on bacterial composition (Figs. 2 and 7), likely due to the dominant influence of biochar. Since *r*-strategists generally have rapid growth and high resource use efficiency, their enrichment may accelerate soil nutrient cycling and affect ecosystem stability. Biochar also significantly reduced the relative abundance of pathogenic bacteria (Fig. 4d). Growing evidence suggests that improvements in soil composition and nutrient dynamics under biochar amendments can strengthen microbial interactions and stabilize community networks (Palansooriya et al. 2025). Co-occurrence network analysis revealed that biochar significantly altered microbial network structure by increasing positive correlations, reducing complexity, and enhancing stability (Fig. 6). These changes suggest biochar fosters mutualistic microbial interactions, improving network robustness (Lei et al. 2023). From a network perspective, Planctomycetota, Bacillota, Cyanobacteriota, and Pseudomonadota emerged as hubs involved in organic matter decomposition, nitrogen cycling, and stress adaptation (Fig. S7; Gao et al. 2024; Kracmarova-Farren et al. 2024; Zhou et al. 2025). This indicates that biochar promotes functional specialization rather than maintaining high diversity and redundancy (de Bello et al. 2021; Dong et al. 2024). Microbial network complexity reflects the strength of taxa correlations, with lower complexity possibly indicating compressed ecological niches due to functional group selection (Guseva et al. 2022). We acknowledge that correlation-based co-occurrence networks do not represent true ecological interactions and should be interpreted as statistical associations rather than causal relationships. Network metrics such as modularity, connectivity, and degree distribution provide useful proxies for potential interaction structure, but they do not represent direct measures of ecological resilience (Deng et al. 2012; Yuan et al. 2021). Recognizing this limitation, we interpret our network results cautiously. In particular, the inverse relationship we observed between reduced network complexity and higher stability should be considered a pattern emerging from coordinated shifts across multiple indices rather than evidence of a universal mechanistic trade-off. The consistent decrease in edges and connectivity, together with a higher proportion of positive correlations and increased robustness, suggests a reorganization of microbial interaction networks under biochar amendment. However, these patterns require targeted experimental validation before causal mechanisms can be established.

Environmental variable analysis identified BD as a key factor affecting network structure and stability, further reinforcing the central premise of hypothesis 3 regarding the role of soil physical properties in microbial community assembly. Biochar amendments have been widely reported to promote beneficial microbes like Pseudomonadota and Bacillota by creating favorable habitats. These taxa can suppress pathogens through resource competition, nutrient limitation, or spatial occupation (Wu et al. 2022). Our findings further show that biochar inhibited bacterial community succession and increased stochasticity (Fig. 5), likely due to its porous composition and microenvironmental stability, which buffer microbial communities from external fluctuations (Lu et al. 2020). Additionally, biochar may gradually release nutrients, supporting long-term conditions for certain microbial ecotypes, reducing succession rates (Dungait et al. 2012). No significant pH effect was observed, possibly because most biochar was alkaline, and the main soil types Mollisol and Luvisol had differing pH buffering capacities (Data S1). This aligns with previous findings that major microbial shifts occur near pH 5.5, which is uncommon in arable soils (Griffiths and Philippot 2013; Jones et al. 2019). Biochar also increased SOC and total nitrogen levels (Fig. 7), which regulate microbial metabolism and interactions (Ma et al. 2022; Zhou et al. 2024). By improving nitrogen availability and refining niches of nitrogen-cycling microbes, biochar may enhance microbial network assembly. However, these effects are likely influenced by biochar properties (e.g., C/N ratio, application rate) and initial soil nutrient conditions.

By integrating machine learning and co-occurrence network analysis, this study provided an in-depth assessment of microbial biomarkers and the ways in which biochar alters community succession and interactive network structures. Biochar substantially reshaped microbial community composition and ecological networks, not by increasing α-diversity but by promoting community reassembly and modifying interaction patterns. These shifts indicate that biochar can influence microbial community stability, although the underlying regulatory mechanisms involve multiple environmental dimensions. While biochar properties contributed to some variation, our model selection results showed that soil bulk density, soil organic carbon, climatic factors, and application duration exerted far stronger effects at a global scale. Future research should further investigate these interacting mechanisms and explore how biochar microbe relationships vary across different climatic regions, soil types, and biochar materials. Additionally, it is unclear how long these physicochemical changes associated with biochar application will be a shaping factor for the biological community. It has been shown that aging of biochar in the environment reduces its specific surface area and surface functionality (Liu and Chen 2022), likely reducing its capacity to retain soil nutrients over time. Equally, physical fragmentation of biochar particles through mechanical tillage as well as exposure to environmental factors (for example, precipitation, temperature and biological interaction) may reduce the influence of biochar as a refugia (de la Rosa et al. 2018). Ultimately these shifts in microbial diversity and function may be transient (< 10 years) unless biochar is reapplied (Jones et al. 2012). Equally, biochar can vary considerably based on feedstock and pyrolysis conditions (He et al. 2024). Often biochar characteristics are underreported in the literature, particularly in soil related studies, making it difficult to unpick the effect of biochar C stability and functionality (atomic H: C ratios and O: C ratios as common proxies), which are often related to durability (Adhikari et al. 2024; Ippolito et al. 2020), on soil microbial communities. Improved reporting of biochar characteristics is recommended in future studies to aid understanding of these interactions. As with any meta-analysis, our results may be affected by uneven study representation, publication bias, and the fact that we considered only pure biochar applications. Nonetheless, the unified reprocessing of raw sequences reduces methodological heterogeneity and provides a consistent basis for identifying cross-study ecological patterns. Biochar appears to reshape bacterial communities by increasing compositional stability while reducing functional complexity. Trait-based analyses also suggest a link between biochar amendment and microbial life-history tendencies, reflected in associations between rrn operon copy number, oligotroph/copiotroph patterns, and application duration. The concurrent increase in stability and decline in network complexity highlights a possible ecological trade-off relevant to long-term soil carbon dynamics. Although these interpretations remain exploratory, they offer a useful framework for future research. Overall, moderate and sustained biochar inputs, combined with balanced nutrient management, may help promote microbial functional stability and soil health.

## Conclusions

This study synthesizes data from 24 independent studies (843 data points) to assess the impact of biochar application on soil bacterial diversity, community composition, and functional strategies. The results show that biochar significantly improves key soil properties but does not have a marked effect on bacterial α-diversity. However, biochar significantly alters bacterial survival strategies and community composition, as evidenced by a decrease in the oligotrophs/copiotrophs ratio and a reduction in the relative abundance of Planctomycetota. Biochar impacts microbial communities primarily through changes in soil properties especially bulk density. Machine learning models identified 138 key bacterial genera as biomarkers. Co-occurrence network analysis revealed that biochar application simplified core microbial networks, increased positive interactions, and enhanced microbial community stability. Overall, biochar amendments drive significant shifts in microbial community composition and interactions, primarily through improvements in soil nutrient status, though further research should aim to understand the longevity of this effect. These findings highlight the role of biochar in enhancing soil stability and influencing bacterial survival strategies, with important implications for sustainable soil management and C sequestration. Future research should examine how specific soil properties regulate microbial responses to biochar and assess the persistence of these effects across longer time scales. Additional field studies are needed to test whether the observed microbial shifts remain consistent across different biochar types and soil environments.

## Supplementary information

Below is the link to the electronic supplementary material.

Supplementary Material 1

Supplementary Material 2

## Data Availability

The data and code that support the findings of this study are openly available in Github at https://github.com/uby76/Biochar
