# Supplementary material for: Biochar reshapes soil bacterial community composition and survival strategies: a meta-analysis revealing trade-offs between microbial stability and functional complexity
Source: Biol Fertil Soils. 2025 Dec 19;62(2):251–66. doi: 10.1007/s00374-025-01971-9 (PMC12855287; doi:10.1007/s00374-025-01971-9)
Supplement: Supplementary file 1 — Supplementary Material 1 [file 374_2025_1971_MOESM1_ESM.docx]

***Supplementary Material***

**Biochar reshapes soil bacterial** **community structure and survival strategies: A meta-analysis revealing trade-offs between microbial stability and functional complexity**

Jianwei Li^a^, Peduruhewa H. Jeewani^b,^ *, Robert W. Brown^b^, David R. Chadwick^b^, Robert I. Griffiths^b^, Haoran Fu^c^, Hongfeng Bian^a^, LianXi Sheng^a^, Qinqxu Ma^c^, Davey L. Jones^b^

^a^ *State Environmental Protection Key Laboratory of Wetland Ecology and Vegetation Restoration, School of Environment, Northeast Normal University, Changchun 130117, China*

^b^ *School of Environmental & Natural Sciences, Bangor University, Bangor LL57 2UW, UK*

^c^ *Zhejiang Provincial Key Laboratory of Agricultural Resources and Environment, College of Environmental and Resource Sciences, Zhejiang University, Hangzhou 310058, China*

**Supplementary Text S1:** Raw sequence data in FASTQ format were downloaded from publicly available databases. Data processing followed the standard operating procedures outlined by Comeau et al. (2017) using QIIME2. Sequence quality was assessed with FastQC v0.11.7, and primers were removed using Cutadapt (Martin, 2011). Paired-end reads were merged using vsearch (Rognes et al., 2016), based on overlapping regions of the paired reads. Reads with a Phred quality score of < 20 were discarded. Low-quality reads were subsequently filtered using the default quality thresholds in QIIME2. Sequences were denoised and assigned to amplicon sequence variants (ASVs) using Deblur (Amir et al., 2017; Wright et al., 2021). For each study, a trimmed sequence length was determined based on the read quality distribution. In some studies, only forward reads were used due to the poor quality of reverse reads, which resulted in a reduced number of sequences after merging and Deblur processing. Data processing was performed individually for each study to ensure thorough and accurate handling of sequencing data. All processed datasets were combined using the Qiime feature-table merge and Qiime feature-table merge-seqs commands in QIIME2. Taxonomic annotation was performed using the SILVA v132 database. Sequences with a cumulative abundance of ≤10 and samples with fewer than 2000 reads were excluded. The data was then diluted to 2,000 reads per sample to account for the wide variation in sequencing depth across samples, and the final dataset included 843 samples from 24 studies. Similar methods have been employed in previous studies, such as Adams et al. (2015) and Lei et al. (2023), which combined sequencing data to examine shifts in microbial community structures. Whilst applying the analytical approach of Wright et al. (2021) to sample rarefaction and calculating relative abundances to mitigate differences in sequencing depth across studies. To enhance the reliability of our findings, we incorporated different studies as random effects in our model, thereby reducing methodological biases stemming from variations in primer selection, PCR conditions, and sequencing platforms. Given the substantial variability at the ASV level, our analysis focused on the genus level while excluding unannotated samples from the database. This approach ensured the retention of taxonomic diversity while addressing the challenges of merging ASVs across different datasets and primers, ultimately improving analytical accuracy (Chong et al., 2020). Additionally, we conducted data analysis at the ASV level, excluding ASVs present in less than 10% of samples to minimize technical artifacts and prevent overestimation of species richness. Notably, our results showed that ASV-level analyses did not enhance classification accuracy between control and biochar-treated groups (Table S3), consistent with previous findings that ASV-based classifications do not significantly improve predictive accuracy (Lei et al., 2023; Wright et al., 2021; Yuan et al., 2020).

**Supplementary** **Text S2**: Machine Learning-Based Classification of Microbial Communities

To further differentiate microbial communities between biochar-treated and control groups, three well-established machine learning algorithms—Random Forest (RF), Support Vector Machine (SVM), and Logistic Regression (LR)—were implemented using the randomForest, e1071, and car packages in R, respectively (Liaw et al., 2002; Meyer et al., 2015; Fox et al., 2012). Model performance was evaluated using ROC curve analysis with the pROC and ROCR packages (Robin et al., 2011; Sing et al., 2005; Yuan et al., 2020). The models were constructed using ASV relative abundances as candidate features, and their performance was assessed through five-fold cross-validation on the training set. Specifically, the training data were randomly split into five equal subsets. In each iteration, Least Absolute Shrinkage and Selection Operator (LASSO) was applied to 80% (674 samples) of the training data for feature selection, and classifiers were trained using the selected features (RF with 500 trees, SVM with a radial basis function kernel, and default settings for other parameters). The trained classifiers were then used to predict or validate the remaining 20% (169 samples) of the training data. The predictions from all five iterations were aggregated, and classification performance was evaluated using the receiver operating characteristic (ROC) curve and the area under the curve (AUC) score. Finally, the best-performing algorithm (with the highest AUC) was selected to build the final classifier using the entire training set. This optimal classifier was then applied to the test set for independent validation of predictive performance. Utilising the randomForest package, the rfcv () function was used to perform cross-validation for feature selection, while the varImpPlot function illustrated the importance of features in classification (Liaw and Wiener, 2002). ROC curves were visualized using the ggplot2 package (Wickham et al., 2016).


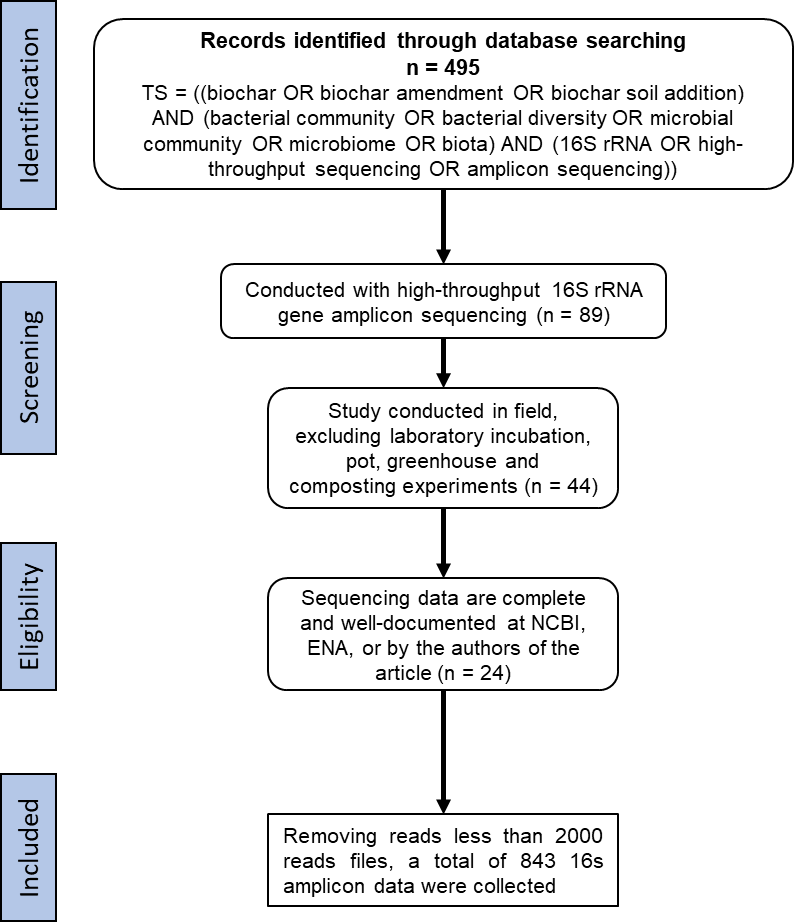


**Fig. S1.** PRISMA flow diagram for the studies selected and included in this meta-analysis. Each article was reviewed to ensure that it met the following criteria: i) experimental design focused on the use of biochar-amended soils, ii) included both control and biochar-treated samples in a paired comparison setup, iii) use of the 16S rRNA gene to detect bacterial diversity and amplification of the V3-V4 or V4 hypervariable region using commonly used primers.


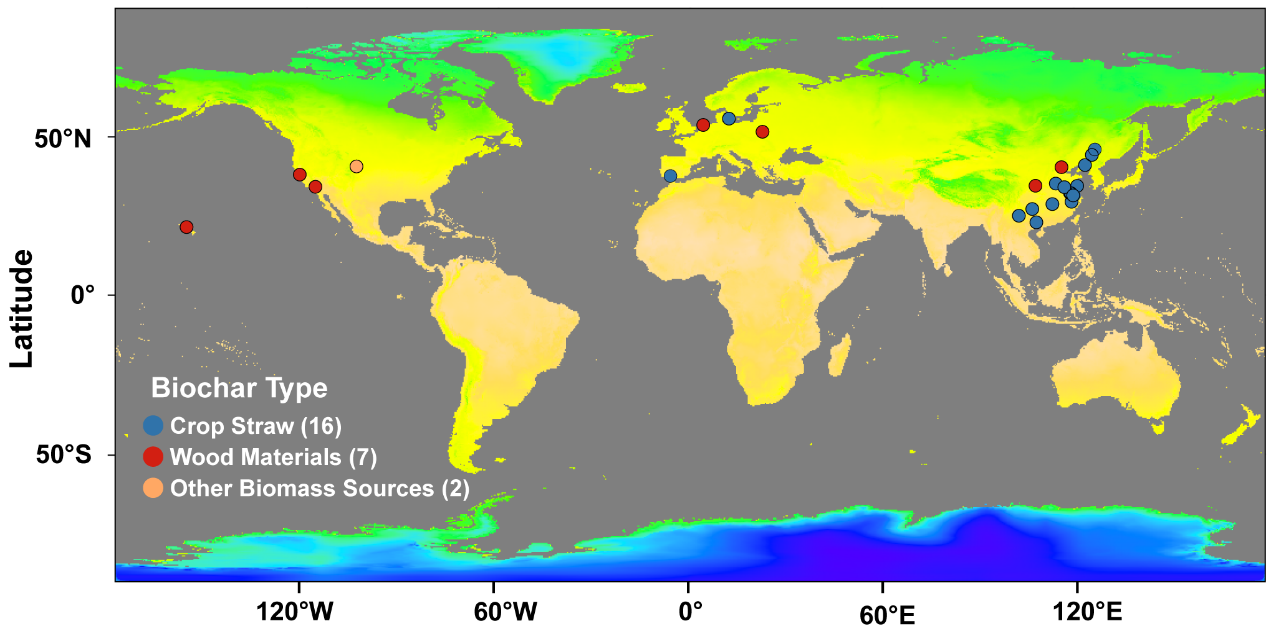


**Fig. S2.** Site locations of the 843 samples from 24 publications included in this study.


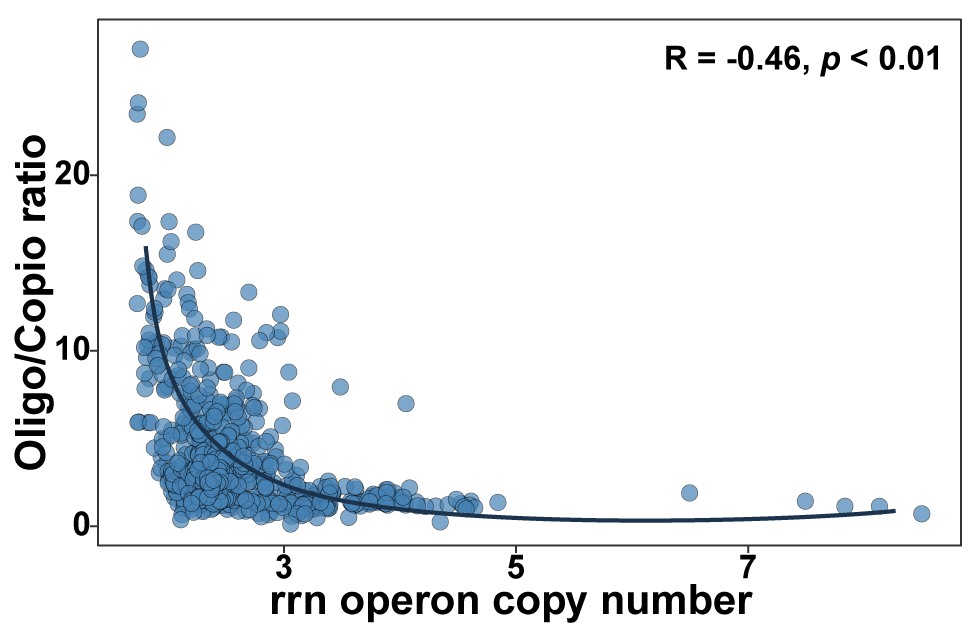


**Fig. S3.** Relationship between the ratio of oligotrophs to copiotrophs (Oligo/Copio ratio) bacteria and the 16S rRNA (rrn) operon copy number of the bacterial community.


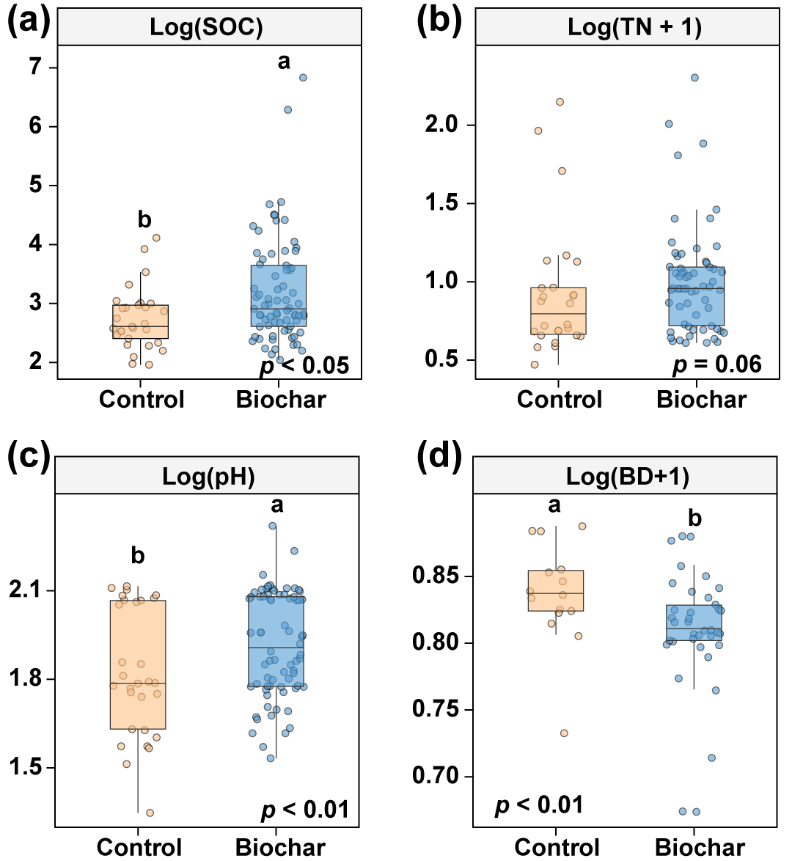


**Fig. S4.** Variations in soil physicochemical indicators across different treatments. Different studies were treated as random effects, and the means were calculated based on different sampling points. The Mann-Whitney U test was used for statistical analysis. In each boxplot, the horizontal line represents the median, while the top and bottom edges denote the 75th and 25th percentiles, respectively.


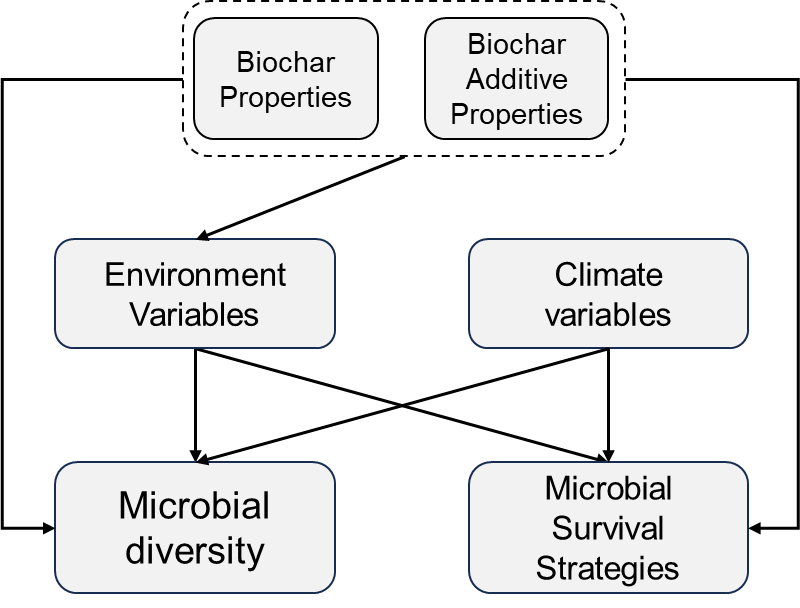


**Fig. S5.** A priori model incorporating all hypothesized pathways was constructed.


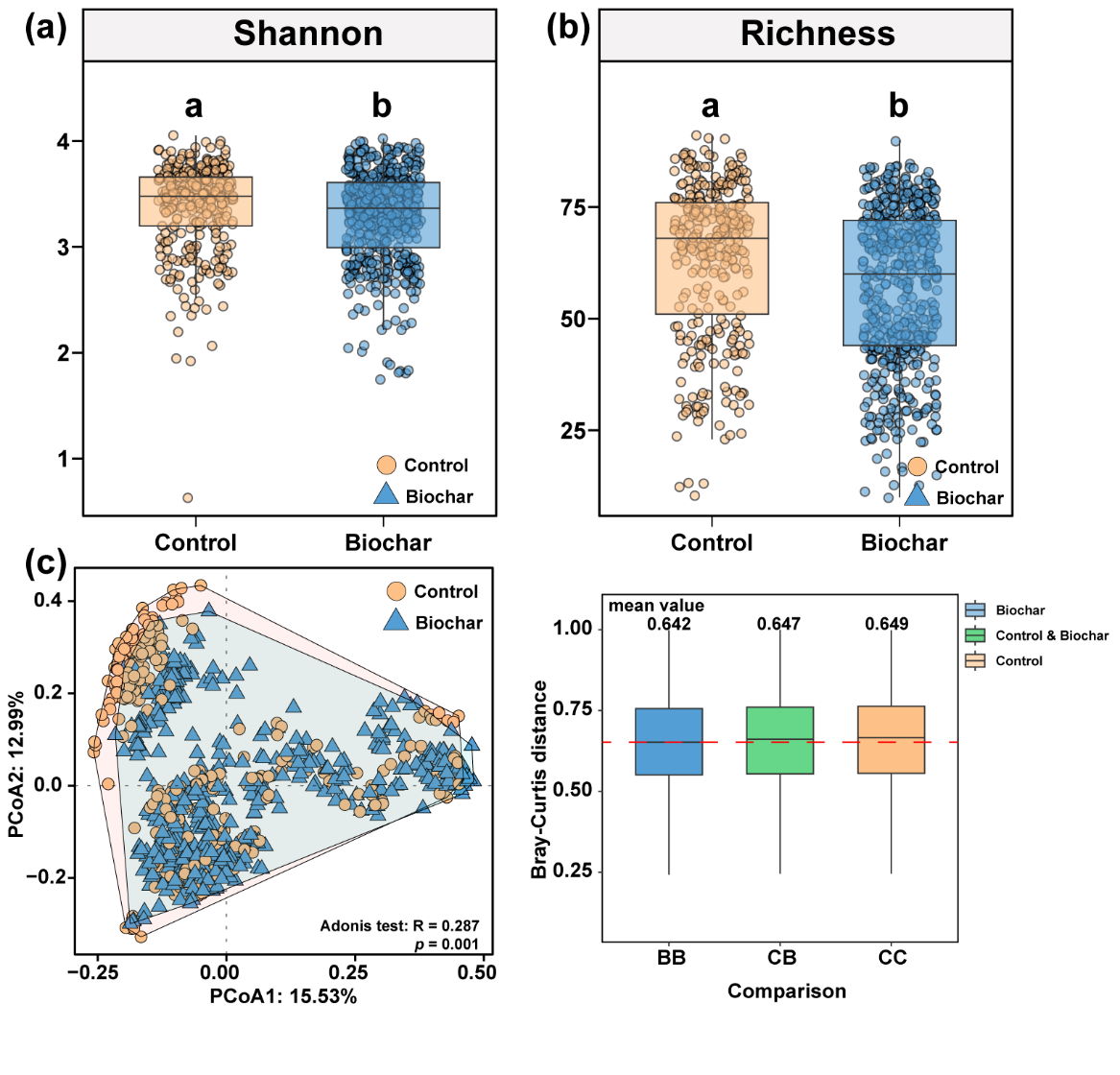


**Fig. S6.** (a,b) α-Diversity (Shannon and Richness) and (c) Principal Coordinate Analysis (PCoA) of core microorganisms, additionally, (d) the distance similarity within and between the biochar and control groups was analyzed. Statistical analysis was conducted using the Mann-Whitney U test. In each boxplot, the horizontal line represents the median, while the top and bottom edges denote the 75th and 25th percentiles, respectively.


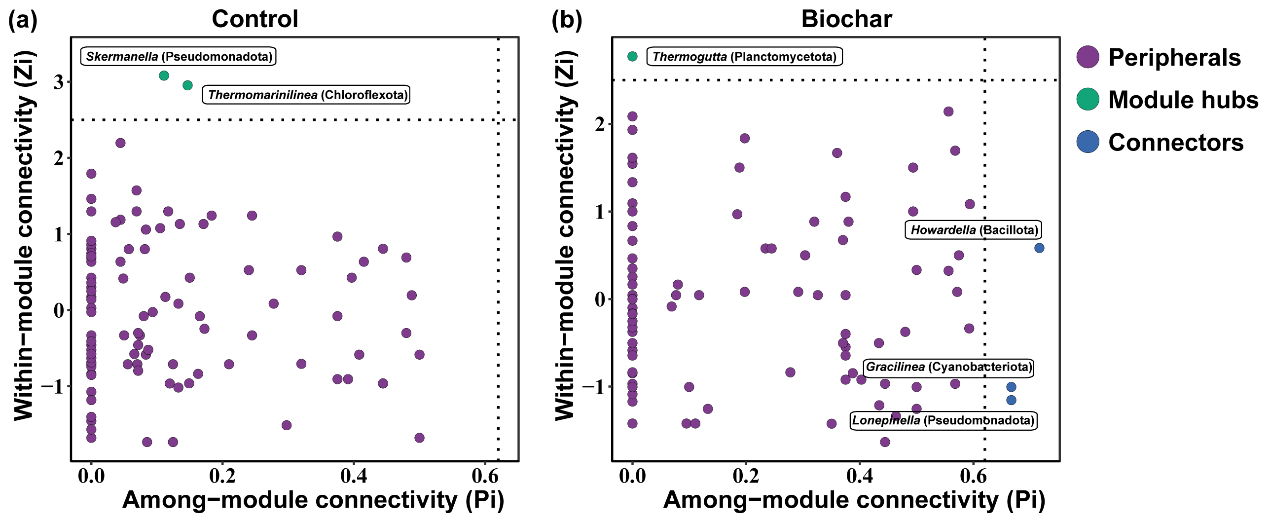


**Fig. S7.** Zi-Pi diagram showing the distribution of keystone species inferred from topological roles.


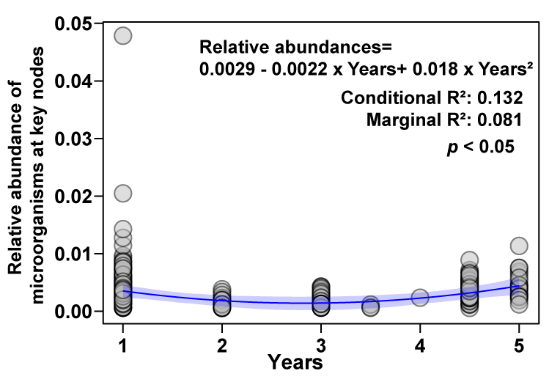


**Fig. S8.** Experiments involving biochar applications of one year or less, as well as those exceeding five years, were excluded. Correlation analyses, modeled using a quadratic function, were performed to examine the relationship between biochar application duration and the relative abundance of key species, with different studies treated as random effects. The R^2^ values were calculated for both the conditional and marginal aspects of the model.

**Table S1.** Relative abundance of species of species at Control and Biochar phylum levels. The p-value calculation was derived from the Mann-Whitney U test to assess whether there was a significant difference between the control and biochar treatments.

| Phylum | P value | Control (%) | Biochar (%) |
| --- | --- | --- | --- |
| Pseudomonadota | 0.513 | 45.42 | 45.75 |
| Actinomycetota | 0.417 | 12.10 | 13.79 |
| Bacillota | 0.417 | 11.55 | 12.15 |
| Bacteroidota | 0.689 | 11.46 | 10.94 |
| Cyanobacteriota | 0.489 | 6.59 | 6.16 |
| **Planctomycetota** | **0.001** | **3.79** | **3.19** |
| Chloroflexota | 0.759 | 3.01 | 2.72 |
| Mycoplasmatota | 0.576 | 3.22 | 2.10 |
| Acidobacteriota | 0.759 | 1.41 | 1.48 |
| Verrucomicrobiota | 0.071 | 0.21 | 0.22 |

**Table S2.** Relative abundance of species in the core microbial control and biochar groups. p-values were calculated from the Mann-Whitney U test to assess whether significant differences existed between the control and biochar treatments.

|  | P value | Control (%) | Biochar (%) |
| --- | --- | --- | --- |
| Acidobacteriota | 0.124 | 10.57 | 6.75 |
| Abditibacteriota | 0.621 | 0.41 | 0.77 |
| Actinomycetota | 0.717 | 8.52 | 7.91 |
| Aquificota | 0.617 | 2.03 | 1.42 |
| **Armatimonadota** | **0.009** | **2.64** | **1.32** |
| Bacillota | 0.308 | 1.91 | 2.18 |
| Bacteroidota | 0.717 | 3.93 | 3.5 |
| **Chlorobiota** | **0.042** | **0.23** | **0.37** |
| **Chloroflexota** | **<0.001** | **10.34** | **9.48** |
| Cyanobacteriota | 0.401 | 0.94 | 1.34 |
| Deinococcota | 0.356 | 0.76 | 0.16 |
| Gemmatimonadota | 0.991 | 18.31 | 22.97 |
| Nitrospirota | 0.485 | 15.89 | 21.06 |
| **Planctomycetota** | **<0.001** | **4.90** | **3.56** |
| **Pseudomonadota** | **0.009** | **3.38** | **3.88** |
| **Spirochaetota** | **0.018** | **5.07** | **2.59** |
| Thermomicrobiota | 0.485 | 9.08 | 10.00 |
| **Verrucomicrobiota** | **<0.001** | **1.09** | **0.74** |

**Table S3.** Results AUC and OOB estimate error rate results for random forest models at ASVs and Genus levels.

|  | AUC | OOB estimate of error rate |
| --- | --- | --- |
| ASVs (ASVs present in at least 10% of samples) | 0.731 | 23.41% |
| Genus | 0.859 | 23.56% |

**Note S1** The studies included in the meta-analysis database.

**1.** Ali, I., Yuan, P., Ullah, S., Iqbal, A., Zhao, Q., Liang, H., Khan, A., Imran, Zhang, H., Wu, X., Wei, S., Gu, M., Jiang, L., 2022. Biochar Amendment and Nitrogen Fertilizer Contribute to the Changes in Soil Properties and Microbial Communities in a Paddy Field. Front. Microbiol. 13. https://doi.org/10.3389/fmicb.2022.834751

**2.** Campos, P., Miller, A.Z., Prats, S.A., Knicker, H., Hagemann, N., De la Rosa, J.M., 2020. Biochar amendment increases bacterial diversity and vegetation cover in trace element-polluted soils: A long-term field experiment. Soil Biology and Biochemistry 150, 108014. https://doi.org/10.1016/j.soilbio.2020.108014

**3.** Cheng, J., Lee, X., Tang, Y., Zhang, Q., 2019. Long-term effects of biochar amendment on rhizosphere and bulk soil microbial communities in a karst region, southwest China. Applied Soil Ecology 140, 126–134. https://doi.org/10.1016/j.apsoil.2019.04.017

**4.** Foster, E.J., Baas, P., Wallenstein, M.D., Cotrufo, M.F., 2020. Precision biochar and inoculum applications shift bacterial community structure and increase specific nutrient availability and maize yield. Applied Soil Ecology 151, 103541. https://doi.org/10.1016/j.apsoil.2020.103541

**5.** Gao, W., Gao, K., Guo, Z., Liu, Y., Jiang, L., Liu, C., Liu, X., Wang, G., 2021. Different Responses of Soil Bacterial and Fungal Communities to 3 Years of Biochar Amendment in an Alkaline Soybean Soil. Front. Microbiol. 12. https://doi.org/10.3389/fmicb.2021.630418

**6.** Hale, L., Curtis, D., Azeem, M., Montgomery, J., Crowley, D.E., McGiffen, M.E., 2021a. Influence of compost and biochar on soil biological properties under turfgrass supplied deficit irrigation. Applied Soil Ecology 168, 104134. https://doi.org/10.1016/j.apsoil.2021.104134

**7.** Hale, L., Curtis, D., Leon, N., McGiffen, M., Wang, D., 2021b. Organic amendments, deficit irrigation, and microbial communities impact extracellular polysaccharide content in agricultural soils. Soil Biology and Biochemistry 162, 108428. https://doi.org/10.1016/j.soilbio.2021.108428

**8.** Han, Z., Xu, P., Li, Z., Lin, H., Zhu, C., Wang, J., Zou, J., 2022. Microbial diversity and the abundance of keystone species drive the response of soil multifunctionality to organic substitution and biochar amendment in a tea plantation. GCB Bioenergy 14, 481–495. https://doi.org/10.1111/gcbb.12926

**9.** Imparato, V., Hansen, V., Santos, S.S., Nielsen, T.K., Giagnoni, L., Hauggaard-Nielsen, H., Johansen, A., Renella, G., Winding, A., 2016. Gasification biochar has limited effects on functional and structural diversity of soil microbial communities in a temperate agroecosystem. Soil Biology and Biochemistry 99, 128–136. https://doi.org/10.1016/j.soilbio.2016.05.004

**10.** Jiang, Z., Yang, S., Pang, Q., Xu, Y., Chen, X., Sun, X., Qi, S., Yu, W., 2021. Biochar improved soil health and mitigated greenhouse gas emission from controlled irrigation paddy field: Insights into microbial diversity. Journal of Cleaner Production 318, 128595. https://doi.org/10.1016/j.jclepro.2021.128595

**11.** Kubaczyński, A., Walkiewicz, A., Pytlak, A., Grządziel, J., Gałązka, A., Brzezińska, M., 2022. Biochar dose determines methane uptake and methanotroph abundance in Haplic Luvisol. Science of The Total Environment 806, 151259. https://doi.org/10.1016/j.scitotenv.2021.151259

**12.** Li, S., Wang, S., Fan, M., Wu, Y., Shangguan, Z., 2020. Interactions between biochar and nitrogen impact soil carbon mineralization and the microbial community. Soil and Tillage Research 196, 104437. https://doi.org/10.1016/j.still.2019.104437

**13.** Li, X., Romanyà, J., Li, N., Xiang, Y., Yang, J., Han, X., 2022. Biochar fertilization effects on soil bacterial community and soil phosphorus forms depends on the application rate. Science of The Total Environment 843, 157022. https://doi.org/10.1016/j.scitotenv.2022.157022

**14.** Liu, X., Li, J., Yu, L., Pan, H., Liu, H., Liu, Y., Di, H., Li, Y., Xu, J., 2018. Simultaneous measurement of bacterial abundance and composition in response to biochar in soybean field soil using 16S rRNA gene sequencing. Land Degradation & Development 29, 2172–2182. https://doi.org/10.1002/ldr.2838

**15.** Qiao, Y., Miao, S., Zhong, X., Zhao, H., Pan, S., 2020. The greatest potential benefit of biochar return on bacterial community structure among three maize-straw products after eight-year field experiment in Mollisols. Applied Soil Ecology 147, 103432. https://doi.org/10.1016/j.apsoil.2019.103432

**16.** Wang, C., Chen, D., Shen, J., Yuan, Q., Fan, F., Wei, W., Li, Y., Wu, J., 2021. Biochar alters soil microbial communities and potential functions 3–4 years after amendment in a double rice cropping system. Agriculture, Ecosystems & Environment 311, 107291. https://doi.org/10.1016/j.agee.2020.107291

**17.** Xu, Y., He, L., Chen, J., Lyu, H., Wang, Y., Yang, L., Yang, S., Liu, Y., 2022. Long-Term Successive Biochar Amendments Alter the Composition and α-Diversity of Bacterial Community of Paddy Soil in Rice-Wheat Rotation. Front. Environ. Sci. 10. https://doi.org/10.3389/fenvs.2022.921766

**18.** Yao, Q., Liu, J., Yu, Z., Li, Y., Jin, J., Liu, X., Wang, G., 2017. Changes of bacterial community compositions after three years of biochar application in a black soil of northeast China. Applied Soil Ecology 113, 11–21. https://doi.org/10.1016/j.apsoil.2017.01.007

**19.** Yu, J., Deem, L.M., Crow, S.E., Deenik, J.L., Penton, C.R., 2018. Biochar application influences microbial assemblage complexity and composition due to soil and bioenergy crop type interactions. Soil Biology and Biochemistry 117, 97–107. https://doi.org/10.1016/j.soilbio.2017.11.017

**20.** Zhang, H., Ma, T., Wang, L., Yu, X., Zhao, X., Gao, W., Van Zwieten, L., Singh, B.P., Li, G., Lin, Q., Chadwick, D.R., Lu, S., Xu, J., Luo, Y., Jones, D.L., Jeewani, P.H., 2024. Distinct biophysical and chemical mechanisms governing sucrose mineralization and soil organic carbon priming in biochar amended soils: evidence from 10 years of field studies. Biochar 6, 52. https://doi.org/10.1007/s42773-024-00327-0

**21.** Zhang, H., Wang, S., Zhang, J., Tian, C., Luo, S., 2021. Biochar application enhances microbial interactions in mega-aggregates of farmland black soil. Soil and Tillage Research 213, 105145. https://doi.org/10.1016/j.still.2021.105145

**22.** Zhang, X., Zhang, Q., Zhan, L., Xu, X., Bi, R., Xiong, Z., 2022. Biochar addition stabilized soil carbon sequestration by reducing temperature sensitivity of mineralization and altering the microbial community in a greenhouse vegetable field. Journal of Environmental Management 313, 114972. https://doi.org/10.1016/j.jenvman.2022.114972

**23.** Zhao, L., Guan, H., Wang, R., Wang, H., Li, Z., Li, W., Xiang, P., Xu, W., 2021. Effects of Tobacco Stem-Derived Biochar on Soil Properties and Bacterial Community Structure under Continuous Cropping of Bletilla striata. J Soil Sci Plant Nutr 21, 1318–1328. https://doi.org/10.1007/s42729-021-00442-y

**24.** Zheng, H., Liu, D., Liao, X., Miao, Y., Li, Y., Li, J., Yuan, J., Chen, Z., Ding, W., 2022. Field-aged biochar enhances soil organic carbon by increasing recalcitrant organic carbon fractions and making microbial communities more conducive to carbon sequestration. Agriculture, Ecosystems & Environment 340, 108177. <https://doi.org/10.1016/j.agee.2022.108177>

**References**

Chong, J., Liu, P., Zhou, G., Xia, J., 2020. Using MicrobiomeAnalyst for comprehensive statistical, functional, and meta-analysis of microbiome data. Nature Protocols 15, 799–821. https://doi.org/10.1038/s41596-019-0264-1

Comeau, A.M., Douglas, G.M., Langille, M.G.I., 2017. Microbiome Helper: a Custom and Streamlined Workflow for Microbiome Research. mSystems 2, 10.1128/msystems.00127-16. https://doi.org/10.1128/msystems.00127-16

Martin, M., 2011. Cutadapt removes adapter sequences from high-throughput sequencing reads. EMBnet.journal 17, 10–12. https://doi.org/10.14806/ej.17.1.200

Rognes, T., Flouri, T., Nichols, B., Quince, C., Mahé, F., 2016. VSEARCH: a versatile open source tool for metagenomics. PeerJ 4, e2584. https://doi.org/10.7717/peerj.2584

Amir, A., McDonald, D., Navas-Molina, J.A., Kopylova, E., Morton, J.T., Zech Xu, Z., Kightley, E.P., Thompson, L.R., Hyde, E.R., Gonzalez, A., Knight, R., 2017. Deblur Rapidly Resolves Single-Nucleotide Community Sequence Patterns. mSystems 2, 10.1128/msystems.00191-16. https://doi.org/10.1128/msystems.00191-16

Wright, R.J., Langille, M.G.I., Walker, T.R., 2021. Food or just a free ride? A meta-analysis reveals the global diversity of the Plastisphere. The ISME Journal 15, 789–806. https://doi.org/10.1038/s41396-020-00814-9

Lei, C., Lu, T., Qian, H., Liu, Y., 2023. Machine learning models reveal how biochar amendment affects soil microbial communities. Biochar 5, 89. https://doi.org/10.1007/s42773-023-00291-1

Yuan, J., Wen, T., Zhang, H., Zhao, M., Penton, C.R., Thomashow, L.S., Shen, Q., 2020. Predicting disease occurrence with high accuracy based on soil macroecological patterns of Fusarium wilt. The ISME Journal 14, 2936–2950. https://doi.org/10.1038/s41396-020-0720-5
